# Crystal structure and near-infrared emission of *trans*-di­chlorido­(di­meth­oxy­phenyl­phosphine)[4,4′,4′′-tris­(meth­oxy­carbon­yl)-2,2′:6′,2′′-terpyridine]­ruthenium(II) monohydrate

**DOI:** 10.1107/S2056989025010862

**Published:** 2026-01-01

**Authors:** Takumi Kinoshita, Hiroshi Segawa

**Affiliations:** a Department of General Systems Studies Graduate School of Arts and Sciences The University of Tokyo 3-8-1 Komaba Meguro-ku Tokyo 153-8902 Japan; b Research Center for Advanced Science and Technology (RCAST) The University of Tokyo 4-6-1 Komaba Meguro-ku Tokyo 153-8904 Japan; Tokyo University of Science, Japan

**Keywords:** crystal structure, ruthenium(II) polypyridyl complex, phosphinite ligand, near-infrared emission

## Abstract

In the title compound, [RuCl_2_(C_21_H_17_N_3_O_6_)(C_8_H_11_O_2_P)]·H_2_O, the Ru^II^ atom is coordinated by three N atoms of a meridionally bound 4,4′,4′′-tris­(meth­oxy­carbon­yl)-2,2′:6′,2′′-terpyridine ligand, a phosphinite P donor and two chloride ligands in a distorted octa­hedral geometry. The Ru—N distances lie in the range 1.996 (2)–2.078 (2) Å, with a Ru—P distance of 2.2879 (9) Å and Ru—Cl distances of 2.3713 (8) and 2.4191 (8) Å; the N—Ru—N bite angles are 78.59 (9) and 79.10 (9)°, with an N—Ru—N angle of 157.30 (9)° within the terpyridine chelate. The methyl ester groups adopt conformations that minimize steric inter­actions with the phosphinite phenyl ring and provide potential anchoring sites in the corresponding carb­oxy­lic acid dye. In the crystal, pairs of complex mol­ecules are linked into discrete hydrogen-bonded dimers by the water mol­ecule of crystallization: one H atom forms an O—H⋯O contact to a methyl carbonyl O atom [H⋯O = 2.17 Å] and the other H atom forms an O—H⋯Cl contact to a *trans* chloride ligand of a neighbouring complex [H⋯Cl = 2.40 Å].

## Abstract 2

In the title Ru^II^ complex, a meridionally bound 4,4′,4′′-tris­(meth­oxy­carbon­yl)terpyridine and a di­meth­oxy­phenyl­phosphine ligand define a distorted *trans*-RuN_3_PCl_2_ octa­hedral coordination environment, and water mol­ecules of crystallization bridge pairs of complex mol­ecules into discrete hydrogen-bonded dimers.

## 1. Chemical context

Ruthenium(II) polypyridyl complexes remain among the most widely studied sensitizers for dye-sensitized solar cells (DSSCs) because they combine intense metal-to-ligand charge-transfer (MLCT) absorption with favourable redox properties and robust synthetic tunability (Grätzel, 2005 ▸; Qin *et al.*, 2012 ▸). Within this family, strongly σ-donating phosphine or phosphinite co-ligands have been used to modulate the ligand field at the Ru^II^ atom, alter the energy and composition of the frontier *d* orbitals and, in some cases, to enable spin-forbidden singlet–triplet excitations that extend the spectroscopic response into the near-infrared region (Kinoshita *et al.*, 2013 ▸; De Angelis, 2014 ▸; Kinoshita *et al.*, 2015 ▸; Swetha *et al.*, 2015 ▸; Kinoshita, 2022 ▸; Juwita *et al.*, 2024 ▸). A notable example is the phosphine-coordinated sensitizer DX1, which employs a tricarb­oxy-substituted terpyridine ligand to anchor onto TiO_2_ and displays an unusually broad photoresponse that has been attributed to spin-forbidden singlet–triplet absorption (Kinoshita *et al.*, 2013 ▸, 2019 ▸; Kinoshita, 2022 ▸). Reliable structural information on such systems is desirable in order to benchmark quantum-chemical calculations, to assess how the strong σ-donor phosphinite perturbs the RuN_3_PCl_2_ coordination environment, and to relate the conformation of the extended π system and the ester groups to the photophysical behaviour of the dye (Fantacci *et al.*, 2014 ▸; Mishima *et al.*, 2015 ▸; Imamura *et al.*, 2015 ▸; Kanno *et al.*, 2016 ▸; Kinoshita *et al.*, 2024 ▸; Juwita *et al.*, 2024 ▸). To date, a single-crystal X-ray structure has been reported only for a thio­phene-extended tricarb­oxy ester analogue (DX4m) (Kinoshita *et al.*, 2021 ▸), and that study focused mainly on the crystal packing rather than on a detailed analysis of the RuN_3_PCl_2_ coordination geometry or the terpyridine conformation in a DX1-type core. The title compound, [RuCl_2_(C_21_H_17_N_3_O_6_)(C_8_H_11_O_2_P)]·H_2_O, is the methyl ester analogue of DX1 and contains a 4,4′,4′′-tris­(meth­oxy­carbon­yl)-2,2′:6′,2″-terpyridine ligand (tcTpy) (Nazeeruddin *et al.*, 2001 ▸; Dehaudt *et al.*, 2011 ▸) and a di­meth­oxy­phenyl­phosphine co-ligand. Its crystal structure therefore provides the first detailed crystallographic insight into the coordination geometry and mol­ecular conformation of a DX1-type Ru^II^ sensitizer.
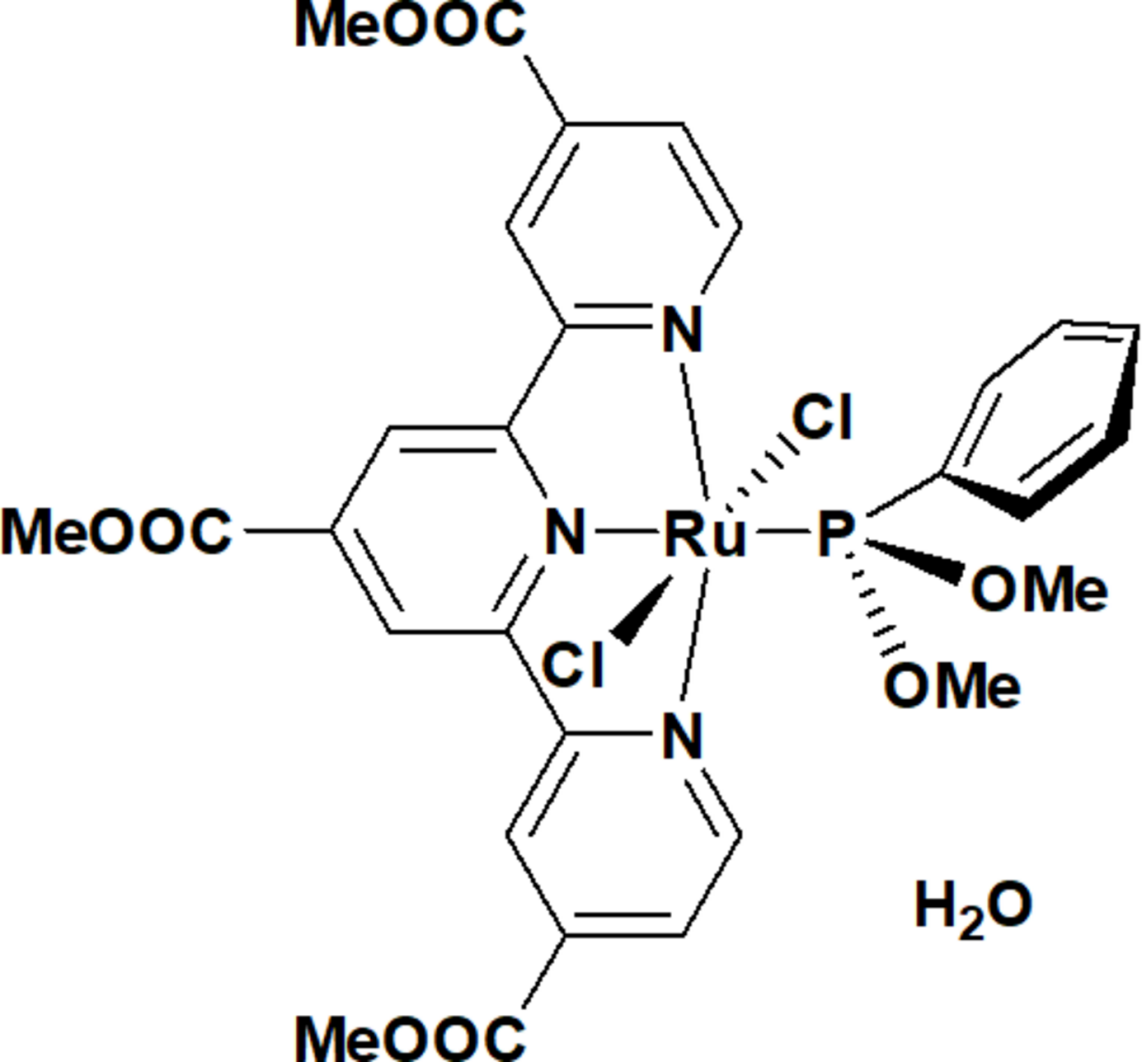


## 2. Structural commentary

**Figure 1:**
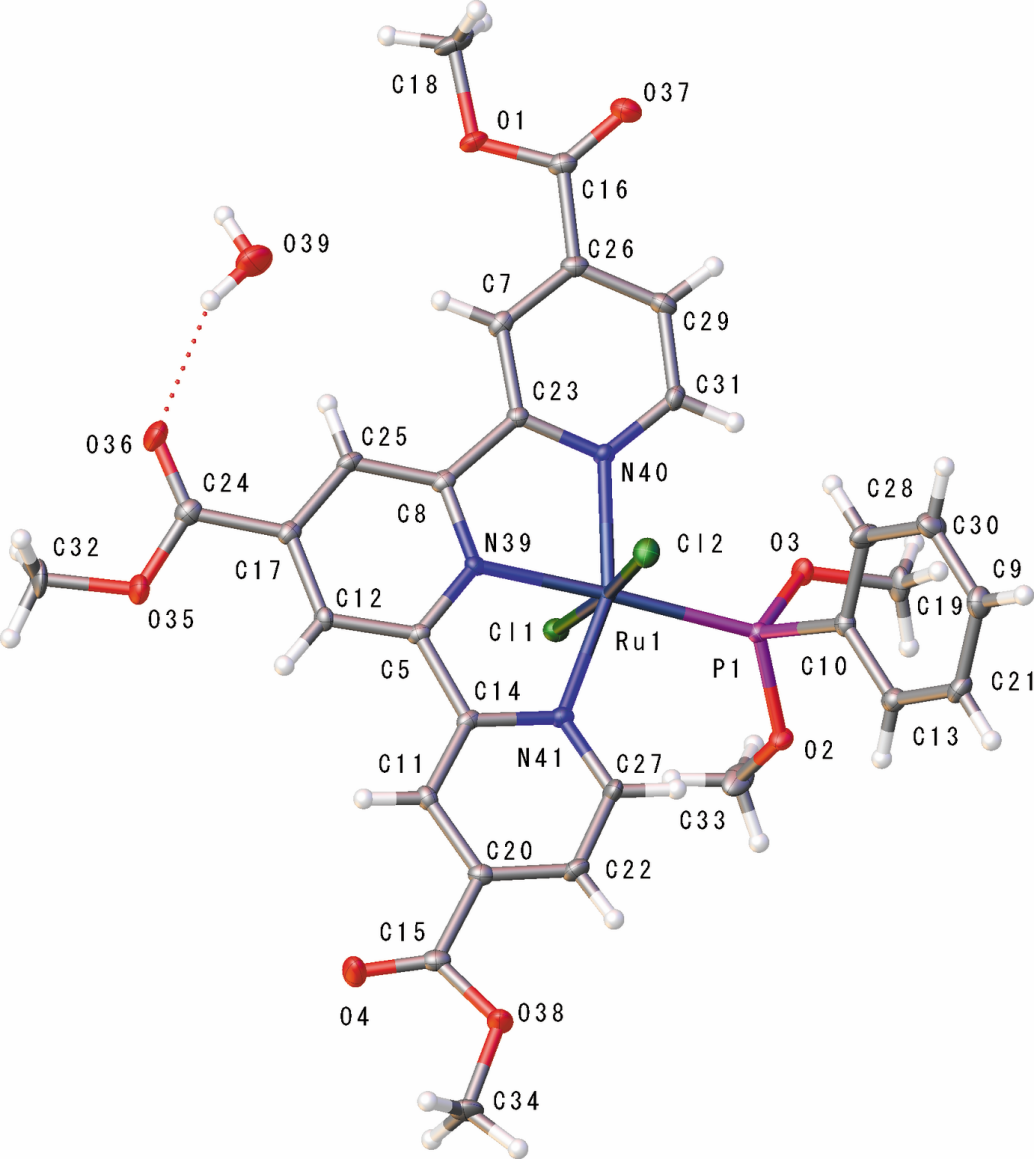
The mol­ecular structure of the title complex, [RuCl_2_(tcTpy)(PPh(OMe)_2_)]·H_2_O, showing the atom-labelling scheme and 50% probability displacement ellipsoids for non-H atoms. The O39—H hydrogen bond is indicated by a dashed line.

The mol­ecular structure of the title complex, [RuCl_2_(tcTpy)(PPh(OMe)_2_)]·H_2_O, is shown in Fig. 1 ▸. The Ru^II^ atom (Ru1) is six-coordinated in a slightly distorted octa­hedral environment defined by three N atoms (N39, N40 and N41) of the meridionally bound tcTpy ligand, one P atom (P1) of the di­meth­oxy­phenyl­phosphine ligand and two chlorido ligands (Cl1 and Cl2). The two Cl ligands occupy mutually *trans* positions [Cl2—Ru1—Cl1 = 174.94 (2)°], while the phosphinite donor P1 is *trans* to the central tcTpy N atom N39 [P1—Ru1—N39 = 177.03 (6)°].

The Ru—N bond lengths are Ru1—N39 = 1.996 (2) Å, Ru1—N40 = 2.078 (2) Å and Ru1—N41 = 2.054 (2) Å, with the shortest distance to the central tcTpy N atom N39. The chelating bite angles within the tcTpy ligand are N39—Ru1—N40 = 79.10 (9)° and N39—Ru1—N41 = 78.59 (9)°, and the corresponding N—Ru—N angle involving the two terminal N donors is N40—Ru1—N41 = 157.30 (9)°, indicating the usual meridional terpyridine binding mode with a modest opening of the N—Ru—N angle opposite the phosphinite ligand. The Ru—P bond length is Ru1—P1 = 2.2879 (9) Å, and the Ru—Cl distances are Ru1—Cl1 = 2.4191 (8) Å and Ru1—Cl2 = 2.3713 (8) Å, all of which are within the ranges commonly observed for Ru^II^ terpyridine–phosphine complexes. The coordination geometry around the Ru^II^ atom can thus be described as a slightly distorted octa­hedron, with the primary distortion arising from the constrained tcTpy bite angles. The tcTpy ligand is close to planar within each pyridyl ring, but the terpyridine unit as a whole shows a slight bowl-shaped curvature, with the terminal rings tilted by about 7.6 and 5.7° with respect to the central ring plane. This curvature most likely reflects steric repulsion between the 4,4′,4′′-meth­oxy­carbonyl substituents and the di­meth­oxy­phenyl­phosphine co-ligand, and it propagates to the metal coordination sphere: as a result, the Cl1—Ru1—Cl2 axis is not exactly perpendicular to the tcTpy mean plane, but is inclined by about 11° from the normal

The three meth­oxy­carbonyl substituents at the 4,4′,4′′ positions are rotated out of the terpyridine mean plane by 1about 0.7, 12.2 and 7.7°, respectively, to relieve steric crowding around the RuN_3_PCl_2_ core and the phosphinite phenyl.The di­meth­oxy­phenyl­phosphine ligand adopts a conformation in which the phenyl ring is roughly orthogonal to the Ru—P bond, while the two meth­oxy groups project away from the metal centre and from the tcTpy core, further minimizing steric congestion around the coordination sphere.

## 3. Supra­molecular features

**Figure 2:**
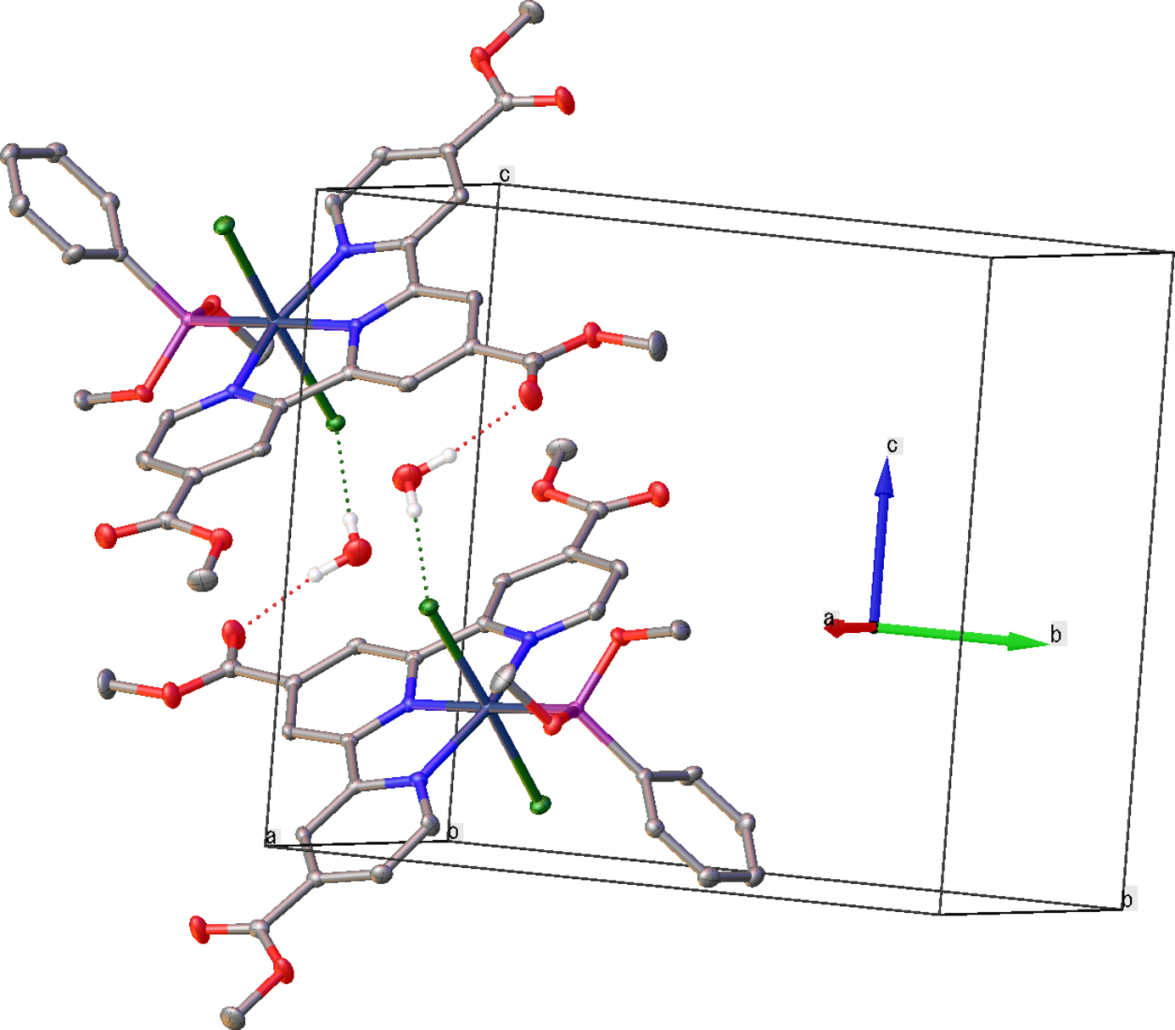
The mol­ecular structure of the title complex with displacement ellipsoids drawn at the 50% probability level. The O39—H39*B*⋯O36 and O39—H39*A*⋯Cl1 hydrogen bonds are indicated by dashed lines; other H atoms are omitted for clarity.

The dominant inter­molecular inter­actions in the crystal are mediated by the water mol­ecule of crystallization (O39), which acts as a hydrogen-bond donor to two adjacent acceptors (Fig. 2 ▸). One H atom, H39*B*, forms an O39—H39*B*⋯O36 hydrogen bond to a methyl­carbonyl O atom of the tcTpy ligand of its own complex, whereas the other H atom, H39*A*, forms an O39—H39*A*⋯Cl1 hydrogen bond to the *trans* chlorido ligand (Cl1) of a neighbouring complex. These contacts link two symmetry-related complexes into a discrete centrosymmetric dimer in which the water mol­ecule bridges between a tcTpy ester group on one mol­ecule and the Ru—Cl fragment of the other. The H⋯O and H⋯Cl separations are 2.17 and 2.40 Å, respectively, and the complete hydrogen-bond geometry is given in Table 1 ▸. Apart from this motif, only relatively long C—H⋯O and C—H⋯Cl contacts are observed between neighbouring mol­ecules, and no extended hydrogen-bonded or π–π-stacked network is formed; the remaining packing is governed mainly by van der Waals contacts between the aromatic ligands.

**Figure 3:**
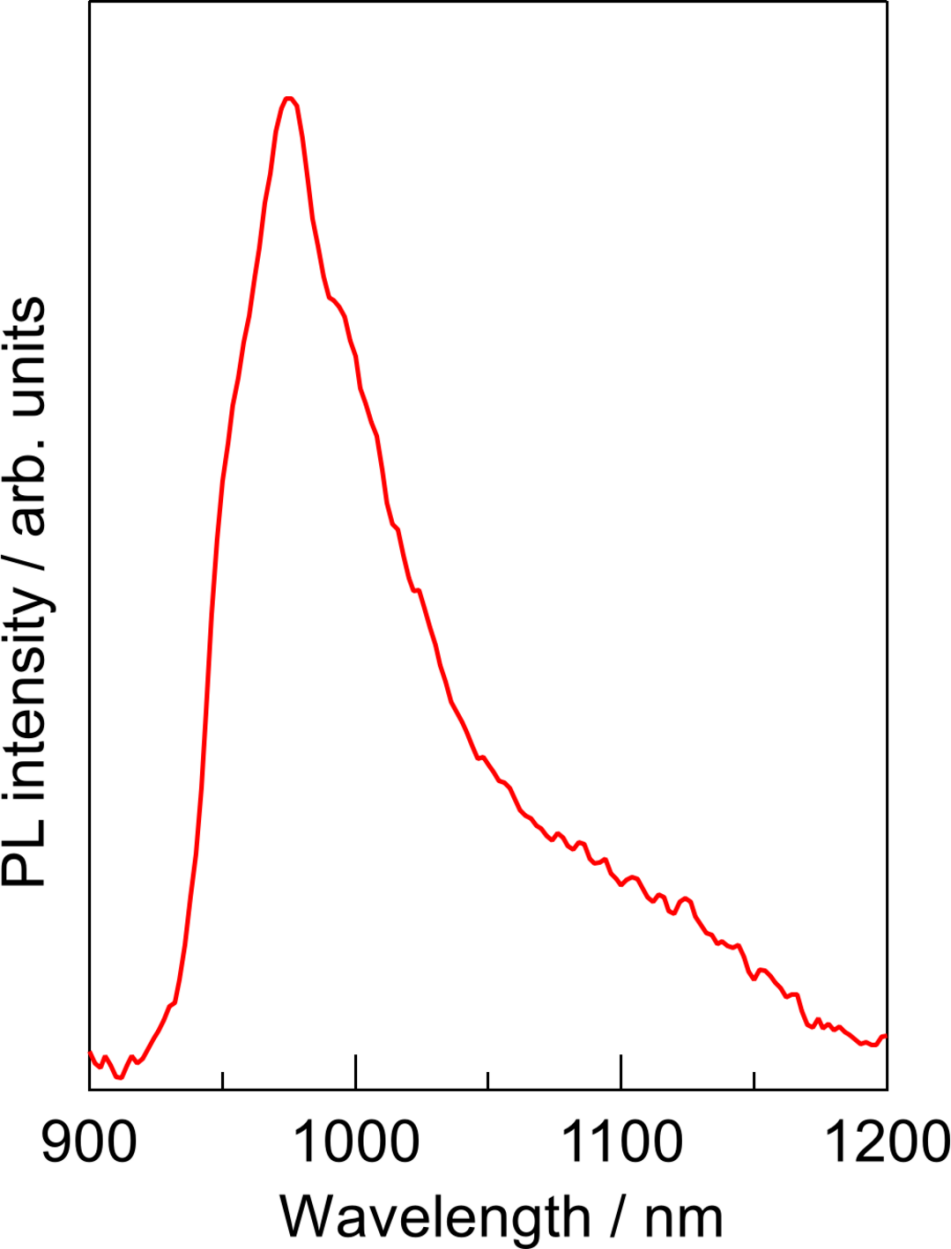
Low-temperature (77 K) single-crystal emission spectrum of the title complex in the near-infrared region (λ_ex_ = 532 nm).

To relate these structural features to the functional behaviour of the complex, low-temperature single-crystal emission measurements at 77 K show a near-infrared band with a maximum around 974 nm, which can be assigned to a Ru^II^→tcTpy triplet MLCT transition (Fig. 3 ▸). The presence of this MLCT-type NIR emission reflects the relatively strong ligand field at the Ru^II^ atom imposed by the meridionally bound tcTpy ligand and the phosphinite ligand trans to its central nitro­gen donor. The band is noticeably sharper than in fluid solution, and its maximum at 974 nm is only slightly red-shifted relative to the solution spectrum, suggesting that crystal-packing effects on the emissive state are modest. This modest perturbation is consistent with the absence of extended π–π stacking and the limited supra­molecular inter­actions beyond the discrete hydrogen-bonded dimers, which restrict inter­molecular quenching pathways and allow the MLCT excited state to be preserved in the solid state.

## 4. Database survey

Inspection of the Cambridge Structural Database (WebCSD, November 2025; Groom *et al.*, 2016 ▸) was carried out for mononuclear Ru^II^ complexes containing the tcTpy ligand, defined here as 4,4′,4′′-tris­(meth­oxy­carbon­yl)-2,2′:6′,2′′-terpyridine coordinated in the usual meridional fashion. In this subset, crystal structures have been reported only in combination with neutral or anionic N-donor or carbon-donor co-ligands (Schulze *et al.*, 2012 ▸; Breivogel *et al.*, 2013 ▸; Yao *et al.*, 2014 ▸; Shao *et al.*, 2015 ▸); no tcTpy complexes bearing a monodentate κ*P*-bound phospho­rus ligand or a terminal halide ligand at the Ru^II^ centre are represented. For comparison, we also searched for mononuclear Ru^II^ complexes with a *trans*-{RuCl_2_(Tpy)(P-donor)} core, defined as an *N*^*N*^*N* terpyridine ligand (parent tpy or 4-substituted derivatives), two mutually *trans* chlorido ligands and a monodentate κ*P*-bound phosphine, phosphinite or phosphite ligand. Under these criteria, only a single crystal structure with a *trans*-{RuCl_2_(tpy-type)(P-donor)} core is currently deposited in the CSD, namely our previously reported thio­phene-extended DX4m complex (CSD refcode EVOQEO; Kinoshita *et al.*, 2021 ▸). Thus, while such *trans*-{RuCl_2_(tpy-type)(P-donor)} species are very rare, no examples incorporating the tcTpy ligand are known, and the title compound represents the first crystallographically characterized Ru^II^ complex of the trans-{RuCl_2_(tcTpy)(P-donor)} type.

## 5. Synthesis and crystallization

The methyl ester analogue of DX1 (DX1m) was prepared according to a published procedure (Kinoshita *et al.*, 2013 ▸). A sample of DX1m was dissolved in di­chloro­methane to give a *ca.* 5 mg mL^−1^ solution, which was filtered and transferred to a small glass tube. Diethyl ether was then carefully layered on top of the CH_2_Cl_2_ solution and allowed to diffuse slowly at room temperature. After several days, black needle-like single crystals of the title complex suitable for X-ray diffraction were obtained from the inter­face between the two solvents.

## 6. Refinement

Crystal data, data collection and structure refinement details are summarized in Table 2 ▸. Hydrogen atoms bonded to carbon were placed in calculated positions and refined using a riding model, with C—H = 0.95–0.99 Å and *U*_iso_(H) = 1.2*U*_eq_(C) for aromatic and methine H atoms, and C—H = 0.98 Å and *U*_iso_(H) = 1.5*U*_eq_(C) for methyl groups; methyl groups were refined as rotating groups. The H atoms of the water mol­ecule of crystallization were located in a difference-Fourier map and refined with restrained O—H and H⋯H distances, with *U*_iso_(H) = 1.5*U*_eq_(O). The largest residual electron-density peak and hole are located in the vicinity of the Ru atom and are not chemically significant.

## Supplementary Material

Crystal structure: contains datablock(s) I. DOI: 10.1107/S2056989025010862/jp2021sup1.cif

Structure factors: contains datablock(s) I. DOI: 10.1107/S2056989025010862/jp2021Isup2.hkl

CCDC reference: 2512964

Additional supporting information:  crystallographic information; 3D view; checkCIF report

Additional supporting information:  crystallographic information; 3D view; checkCIF report

## Figures and Tables

**Table 1:** Hydrogen-bond geometry (Å, °)

*D*—H⋯*A*	*D*—H	H⋯*A*	*D*⋯*A*	*D*—H⋯*A*
O39—H39*B*⋯O36	0.90	2.17	3.00	154.2
O39—H39*A*⋯Cl1^i^	0.85	2.40	3.24	176.3

Symmetry code: (i) 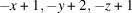.

**Table 2:** Experimental details

Crystal data	
Chemical formula	[Ru(C_8_H_11_O_2_P)Cl_2_(C_21_H_17_N_3_O_6_)]·H_2_O
*M*_r_	767.50
Crystal system, space group	Triclinic, *P* 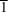
Temperature (K)	273
*a*, *b*, *c* (Å)	10.472 (4), 12.777 (4), 12.973 (4)
α, β, γ (°)	92.254 (3), 108.118 (3), 105.483 (4)
*V* (Å^3^)	1575.7 (9)
*Z*	2
Radiation type	Mo *K*α
μ (mm^−1^)	0.78
Crystal size (mm)	0.11 × 0.04 × 0.03

Data collection	
Diffractometer	Rigaku Saturn724+ (4x4 bin mode)
Absorption correction	Multi-scan (*REQAB*; Rigaku, 2008 ▸)
*T*_min_, *T*_max_	0.900, 0.977
No. of measured, independent and observed [*I* > 2σ(*I*)] reflections	12392, 6845, 5862
*R*_int_	0.029
(sin θ/λ)_max_ (Å^−1^)	0.649

Refinement	
*R*[*F*^2^ > 2σ(*F*^2^)], *wR*(*F*^2^), *S*	0.037, 0.078, 1.05
No. of reflections	6845
No. of parameters	414
H-atom treatment	H atoms treated by a mixture of independent and constrained refinement
Δρ_max_, Δρ_min_ (e Å^−3^)	0.48, −0.98

Computer programs: *CrysAlis PRO* (Rigaku OD, 2025 ▸), *SIR2011* (Burla *et al.*, 2012 ▸), *SHELXL* 2018/3 (Sheldrick, 2015 ▸) and *OLEX2* (Dolomanov *et al.*, 2009 ▸).

## supplementary crystallographic information

### trans-Dichlorido(dimethoxyphenylphosphine)[4,4',4''-tris(methoxycarbonyl)-2,2':6',2''-terpyridine]ruthenium(II) monohydrate Crystal data

**Table:**

[Ru(C_8_H_11_O_2_P)Cl_2_(C_21_H_17_N_3_O_6_)]·H_2_O	*Z* = 2
*M**_r_* = 767.50	*F*(000) = 780
Triclinic, *P*1	*D*_x_ = 1.618 Mg m^−^^3^
*a* = 10.472 (4) Å	Mo *K*α radiation, λ = 0.71075 Å
*b* = 12.777 (4) Å	Cell parameters from 4786 reflections
*c* = 12.973 (4) Å	θ = 3.0–27.5°
α = 92.254 (3)°	µ = 0.78 mm^−^^1^
β = 108.118 (3)°	*T* = 273 K
γ = 105.483 (4)°	Prism, colourless
*V* = 1575.7 (9) Å^3^	0.11 × 0.04 × 0.03 mm

### trans-Dichlorido(dimethoxyphenylphosphine)[4,4',4''-tris(methoxycarbonyl)-2,2':6',2''-terpyridine]ruthenium(II) monohydrate Data collection

**Table:**

Rigaku Saturn724+ (4x4 bin mode) diffractometer	5862 reflections with *I* > 2σ(*I*)
Detector resolution: 7.1429 pixels mm^-1^	*R*_int_ = 0.029
ω scans	θ_max_ = 27.5°, θ_min_ = 3.0°
Absorption correction: multi-scan (*REQAB*; Rigaku, 2008)	*h* = −12→13
*T*_min_ = 0.900, *T*_max_ = 0.977	*k* = −15→16
12392 measured reflections	*l* = −16→16
6845 independent reflections	

### trans-Dichlorido(dimethoxyphenylphosphine)[4,4',4''-tris(methoxycarbonyl)-2,2':6',2''-terpyridine]ruthenium(II) monohydrate Refinement

**Table:**

Refinement on *F*^2^	0 restraints
Least-squares matrix: full	Hydrogen site location: mixed
*R*[*F*^2^ > 2σ(*F*^2^)] = 0.037	H atoms treated by a mixture of independent and constrained refinement
*wR*(*F*^2^) = 0.078	*w* = 1/[σ^2^(*F*_o_^2^) + (0.0275*P*)^2^ + 1.6521*P*] where *P* = (*F*_o_^2^ + 2*F*_c_^2^)/3
*S* = 1.05	(Δ/σ)_max_ = 0.002
6845 reflections	Δρ_max_ = 0.48 e Å^−^^3^
414 parameters	Δρ_min_ = −0.98 e Å^−^^3^

### trans-Dichlorido(dimethoxyphenylphosphine)[4,4',4''-tris(methoxycarbonyl)-2,2':6',2''-terpyridine]ruthenium(II) monohydrate Special details

**Table:**

Geometry. All esds (except the esd in the dihedral angle between two l.s. planes) are estimated using the full covariance matrix. The cell esds are taken into account individually in the estimation of esds in distances, angles and torsion angles; correlations between esds in cell parameters are only used when they are defined by crystal symmetry. An approximate (isotropic) treatment of cell esds is used for estimating esds involving l.s. planes.
Refinement. The structure was solved by direct methods with SIR2011 and refined by full-matrix least squares on F^2^ using SHELXL (Burla *et al.*, 2012) (Sheldrick, 2015). All non-hydrogen atoms were refined with anisotropic displacement parameters.

### trans-Dichlorido(dimethoxyphenylphosphine)[4,4',4''-tris(methoxycarbonyl)-2,2':6',2''-terpyridine]ruthenium(II) monohydrate Fractional atomic coordinates and isotropic or equivalent isotropic displacement parameters (Å^2^)

**Table:**

	*x*	*y*	*z*	*U*_iso_*/*U*_eq_	
Ru1	0.26693 (2)	0.75898 (2)	0.76612 (2)	0.00940 (6)	
Cl1	0.10372 (6)	0.81480 (5)	0.61763 (5)	0.01324 (13)	
Cl2	0.44238 (7)	0.71836 (5)	0.91122 (5)	0.01578 (14)	
P1	0.11296 (7)	0.58928 (6)	0.75016 (5)	0.01076 (14)	
O1	0.6970 (2)	0.81833 (16)	0.44551 (16)	0.0178 (4)	
O2	−0.03404 (19)	0.58173 (15)	0.77135 (15)	0.0136 (4)	
O3	0.0654 (2)	0.51917 (15)	0.63157 (15)	0.0147 (4)	
O4	0.2132 (2)	1.14384 (17)	1.14372 (18)	0.0265 (5)	
O35	0.5711 (2)	1.30809 (16)	0.80683 (17)	0.0204 (4)	
O36	0.6939 (2)	1.25636 (16)	0.71037 (18)	0.0261 (5)	
O37	0.6454 (2)	0.63475 (17)	0.43124 (17)	0.0220 (5)	
O38	0.1028 (2)	0.98566 (16)	1.18854 (17)	0.0224 (5)	
N39	0.3927 (2)	0.91008 (17)	0.77744 (17)	0.0099 (4)	
N40	0.3718 (2)	0.73341 (18)	0.65974 (17)	0.0107 (4)	
N41	0.2247 (2)	0.84184 (18)	0.88451 (17)	0.0115 (4)	
C5	0.3846 (3)	0.9919 (2)	0.8413 (2)	0.0105 (5)	
C7	0.5464 (3)	0.8264 (2)	0.5828 (2)	0.0130 (5)	
H7	0.605083	0.891751	0.574081	0.016*	
C8	0.4690 (3)	0.9284 (2)	0.7096 (2)	0.0118 (5)	
C9	0.2509 (3)	0.3439 (2)	0.9794 (2)	0.0186 (6)	
H9	0.279488	0.294123	1.025057	0.022*	
C10	0.1647 (3)	0.4936 (2)	0.8425 (2)	0.0120 (5)	
C11	0.2783 (3)	1.0195 (2)	0.9870 (2)	0.0117 (5)	
H11	0.323981	1.094341	0.999595	0.014*	
C12	0.4567 (3)	1.0995 (2)	0.8393 (2)	0.0115 (5)	
H12	0.452975	1.156562	0.884038	0.014*	
C13	0.0967 (3)	0.4548 (2)	0.9162 (2)	0.0143 (5)	
H13	0.021542	0.478658	0.919836	0.017*	
C14	0.2931 (3)	0.9527 (2)	0.9071 (2)	0.0107 (5)	
C15	0.1730 (3)	1.0460 (2)	1.1314 (2)	0.0146 (6)	
C16	0.6331 (3)	0.7202 (2)	0.4641 (2)	0.0154 (6)	
C17	0.5348 (3)	1.1204 (2)	0.7687 (2)	0.0127 (5)	
C18	0.7931 (3)	0.8219 (3)	0.3847 (2)	0.0238 (7)	
H18A	0.833338	0.896435	0.375922	0.029*	
H18B	0.866577	0.792261	0.423871	0.029*	
H18C	0.742827	0.779332	0.313966	0.029*	
C19	0.2771 (3)	0.4562 (2)	0.8382 (2)	0.0178 (6)	
H19	0.323914	0.481674	0.789706	0.021*	
C20	0.1946 (3)	0.9736 (2)	1.0481 (2)	0.0126 (5)	
C21	0.1401 (3)	0.3811 (2)	0.9842 (2)	0.0168 (6)	
H21	0.094324	0.356359	1.033467	0.020*	
C22	0.1310 (3)	0.8609 (2)	1.0290 (2)	0.0155 (6)	
H22	0.077372	0.827601	1.070490	0.019*	
C23	0.4629 (3)	0.8268 (2)	0.6475 (2)	0.0111 (5)	
C24	0.6104 (3)	1.2341 (2)	0.7585 (2)	0.0159 (6)	
C25	0.5422 (3)	1.0340 (2)	0.7035 (2)	0.0129 (5)	
H25	0.595205	1.047261	0.657045	0.015*	
C26	0.5412 (3)	0.7274 (2)	0.5312 (2)	0.0140 (5)	
C27	0.1487 (3)	0.7992 (2)	0.9479 (2)	0.0154 (6)	
H27	0.105727	0.723964	0.936109	0.018*	
C28	−0.0272 (3)	0.4072 (2)	0.6091 (2)	0.0202 (6)	
H28A	−0.044857	0.377658	0.535097	0.024*	
H28B	0.016741	0.363179	0.658489	0.024*	
H28C	−0.114471	0.406906	0.618844	0.024*	
C29	0.4494 (3)	0.6319 (2)	0.5438 (2)	0.0153 (6)	
H29	0.444090	0.564498	0.510108	0.018*	
C30	0.3193 (3)	0.3817 (2)	0.9055 (2)	0.0200 (6)	
H30	0.393494	0.356678	0.901450	0.024*	
C31	0.3659 (3)	0.6376 (2)	0.6070 (2)	0.0136 (5)	
H31	0.303253	0.573209	0.613413	0.016*	
C32	0.6376 (3)	1.4223 (2)	0.8022 (3)	0.0264 (7)	
H32A	0.601879	1.468134	0.839231	0.032*	
H32B	0.737438	1.439128	0.837117	0.032*	
H32C	0.617604	1.435000	0.727169	0.032*	
C33	−0.1313 (3)	0.6341 (3)	0.7058 (3)	0.0254 (7)	
H33A	−0.211998	0.621524	0.729132	0.030*	
H33B	−0.086307	0.711477	0.714307	0.030*	
H33C	−0.160483	0.604241	0.630237	0.030*	
C34	0.0660 (3)	1.0450 (3)	1.2671 (2)	0.0232 (7)	
H34A	0.015956	0.993960	1.303651	0.028*	
H34B	0.150140	1.092666	1.319989	0.028*	
H34C	0.007692	1.087802	1.229786	0.028*	
O39	0.7618 (3)	1.1027 (2)	0.5693 (2)	0.0318 (6)	
H39A	0.800 (4)	1.127 (3)	0.523 (3)	0.044 (12)*	
H39B	0.767 (4)	1.163 (3)	0.610 (3)	0.048 (13)*	

### trans-Dichlorido(dimethoxyphenylphosphine)[4,4',4''-tris(methoxycarbonyl)-2,2':6',2''-terpyridine]ruthenium(II) monohydrate Atomic displacement parameters (Å^2^)

**Table:**

	*U*^11^	*U*^22^	*U*^33^	*U*^12^	*U*^13^	*U*^23^
Ru1	0.00922 (10)	0.00957 (12)	0.00963 (10)	0.00205 (8)	0.00404 (8)	0.00137 (8)
Cl1	0.0132 (3)	0.0157 (3)	0.0118 (3)	0.0058 (3)	0.0041 (2)	0.0032 (2)
Cl2	0.0129 (3)	0.0169 (4)	0.0142 (3)	0.0038 (3)	0.0003 (3)	0.0033 (3)
P1	0.0107 (3)	0.0108 (4)	0.0114 (3)	0.0030 (3)	0.0046 (3)	0.0018 (3)
O1	0.0184 (10)	0.0198 (11)	0.0202 (10)	0.0045 (9)	0.0139 (9)	0.0041 (8)
O2	0.0105 (9)	0.0143 (10)	0.0176 (10)	0.0048 (8)	0.0054 (8)	0.0044 (8)
O3	0.0173 (10)	0.0114 (10)	0.0119 (9)	−0.0004 (8)	0.0044 (8)	−0.0014 (7)
O4	0.0421 (14)	0.0127 (12)	0.0300 (12)	0.0031 (10)	0.0241 (11)	−0.0007 (9)
O35	0.0239 (11)	0.0109 (10)	0.0261 (11)	0.0000 (9)	0.0124 (9)	0.0002 (8)
O36	0.0307 (12)	0.0153 (11)	0.0349 (13)	−0.0021 (9)	0.0223 (11)	0.0019 (9)
O37	0.0265 (11)	0.0211 (12)	0.0253 (11)	0.0103 (9)	0.0153 (9)	0.0023 (9)
O38	0.0333 (12)	0.0153 (11)	0.0263 (11)	0.0055 (9)	0.0223 (10)	0.0014 (9)
N39	0.0103 (10)	0.0100 (11)	0.0102 (10)	0.0045 (9)	0.0030 (9)	0.0013 (8)
N40	0.0112 (10)	0.0114 (12)	0.0094 (10)	0.0027 (9)	0.0038 (9)	0.0014 (9)
N41	0.0110 (10)	0.0137 (12)	0.0100 (10)	0.0037 (9)	0.0038 (9)	0.0014 (9)
C5	0.0086 (12)	0.0133 (14)	0.0091 (12)	0.0026 (10)	0.0027 (10)	0.0012 (10)
C7	0.0112 (12)	0.0156 (14)	0.0102 (12)	0.0022 (11)	0.0021 (10)	0.0037 (10)
C8	0.0084 (12)	0.0148 (14)	0.0111 (12)	0.0030 (10)	0.0021 (10)	0.0026 (10)
C9	0.0210 (14)	0.0151 (15)	0.0185 (14)	0.0051 (12)	0.0045 (12)	0.0054 (11)
C10	0.0121 (12)	0.0107 (14)	0.0114 (12)	0.0024 (10)	0.0022 (10)	0.0011 (10)
C11	0.0121 (12)	0.0101 (14)	0.0113 (12)	0.0034 (10)	0.0016 (10)	−0.0002 (10)
C12	0.0120 (12)	0.0092 (13)	0.0111 (12)	0.0013 (10)	0.0026 (10)	−0.0003 (10)
C13	0.0132 (13)	0.0140 (14)	0.0150 (13)	0.0024 (11)	0.0053 (11)	0.0007 (11)
C14	0.0088 (12)	0.0139 (14)	0.0103 (12)	0.0040 (10)	0.0034 (10)	0.0044 (10)
C15	0.0119 (12)	0.0199 (16)	0.0135 (13)	0.0063 (11)	0.0048 (11)	0.0018 (11)
C16	0.0132 (13)	0.0202 (16)	0.0135 (13)	0.0050 (11)	0.0053 (11)	0.0017 (11)
C17	0.0109 (12)	0.0141 (14)	0.0133 (13)	0.0041 (11)	0.0036 (10)	0.0043 (10)
C18	0.0194 (15)	0.0362 (19)	0.0223 (16)	0.0075 (14)	0.0158 (13)	0.0083 (13)
C19	0.0194 (14)	0.0227 (16)	0.0167 (14)	0.0089 (12)	0.0102 (12)	0.0096 (12)
C20	0.0117 (12)	0.0152 (14)	0.0114 (12)	0.0050 (11)	0.0038 (10)	0.0011 (10)
C21	0.0161 (13)	0.0162 (15)	0.0166 (14)	0.0000 (11)	0.0072 (11)	0.0039 (11)
C22	0.0152 (13)	0.0183 (15)	0.0150 (13)	0.0032 (11)	0.0090 (11)	0.0040 (11)
C23	0.0104 (12)	0.0120 (14)	0.0113 (12)	0.0035 (10)	0.0039 (10)	0.0014 (10)
C24	0.0145 (13)	0.0181 (15)	0.0138 (13)	0.0031 (11)	0.0041 (11)	0.0024 (11)
C25	0.0112 (12)	0.0172 (15)	0.0121 (12)	0.0042 (11)	0.0061 (10)	0.0049 (10)
C26	0.0128 (12)	0.0184 (15)	0.0125 (13)	0.0063 (11)	0.0052 (11)	0.0022 (11)
C27	0.0159 (13)	0.0107 (14)	0.0196 (14)	−0.0009 (11)	0.0100 (11)	0.0009 (11)
C28	0.0251 (15)	0.0117 (15)	0.0163 (14)	−0.0014 (12)	0.0029 (12)	−0.0014 (11)
C29	0.0165 (13)	0.0166 (15)	0.0148 (13)	0.0077 (11)	0.0057 (11)	−0.0005 (11)
C30	0.0232 (15)	0.0204 (16)	0.0223 (15)	0.0125 (13)	0.0102 (13)	0.0059 (12)
C31	0.0136 (13)	0.0112 (14)	0.0158 (13)	0.0023 (11)	0.0057 (11)	0.0043 (11)
C32	0.0299 (17)	0.0077 (15)	0.0361 (18)	−0.0034 (13)	0.0115 (15)	0.0004 (13)
C33	0.0145 (14)	0.0282 (18)	0.0385 (19)	0.0119 (13)	0.0096 (14)	0.0182 (14)
C34	0.0298 (16)	0.0267 (17)	0.0218 (15)	0.0105 (14)	0.0189 (14)	0.0025 (13)
O39	0.0415 (15)	0.0330 (15)	0.0318 (14)	0.0118 (12)	0.0258 (12)	0.0120 (11)

### trans-Dichlorido(dimethoxyphenylphosphine)[4,4',4''-tris(methoxycarbonyl)-2,2':6',2''-terpyridine]ruthenium(II) monohydrate Geometric parameters (Å, º)

**Table:**

Ru1—Cl1	2.4191 (8)	C11—C20	1.391 (4)
Ru1—Cl2	2.3713 (8)	C12—H12	0.9300
Ru1—P1	2.2879 (9)	C12—C17	1.396 (4)
Ru1—N39	1.996 (2)	C13—H13	0.9300
Ru1—N40	2.078 (2)	C13—C21	1.384 (4)
Ru1—N41	2.054 (2)	C15—C20	1.503 (4)
P1—O2	1.6250 (19)	C16—C26	1.502 (4)
P1—O3	1.6107 (19)	C17—C24	1.490 (4)
P1—C10	1.814 (3)	C17—C25	1.399 (4)
O1—C16	1.324 (3)	C18—H18A	0.9600
O1—C18	1.452 (3)	C18—H18B	0.9600
O2—C33	1.442 (3)	C18—H18C	0.9600
O3—C28	1.457 (3)	C19—H19	0.9300
O4—C15	1.194 (3)	C19—C30	1.384 (4)
O35—C24	1.333 (3)	C20—C22	1.392 (4)
O35—C32	1.452 (3)	C21—H21	0.9300
O36—C24	1.207 (3)	C22—H22	0.9300
O37—C16	1.210 (3)	C22—C27	1.377 (4)
O38—C15	1.329 (3)	C25—H25	0.9300
O38—C34	1.450 (3)	C26—C29	1.389 (4)
N39—C5	1.345 (3)	C27—H27	0.9300
N39—C8	1.349 (3)	C28—H28A	0.9600
N40—C23	1.364 (3)	C28—H28B	0.9600
N40—C31	1.356 (3)	C28—H28C	0.9600
N41—C14	1.378 (3)	C29—H29	0.9300
N41—C27	1.348 (3)	C29—C31	1.384 (4)
C5—C12	1.386 (4)	C30—H30	0.9300
C5—C14	1.478 (3)	C31—H31	0.9300
C7—H7	0.9300	C32—H32A	0.9600
C7—C23	1.389 (4)	C32—H32B	0.9600
C7—C26	1.386 (4)	C32—H32C	0.9600
C8—C23	1.476 (4)	C33—H33A	0.9600
C8—C25	1.382 (4)	C33—H33B	0.9600
C9—H9	0.9300	C33—H33C	0.9600
C9—C21	1.383 (4)	C34—H34A	0.9600
C9—C30	1.394 (4)	C34—H34B	0.9600
C10—C13	1.392 (4)	C34—H34C	0.9600
C10—C19	1.399 (4)	O39—H39A	0.85 (4)
C11—H11	0.9300	O39—H39B	0.90 (4)
C11—C14	1.389 (4)		
			
Cl2—Ru1—Cl1	174.94 (2)	C12—C17—C25	120.3 (2)
P1—Ru1—Cl1	93.19 (3)	C25—C17—C24	117.9 (2)
P1—Ru1—Cl2	91.78 (3)	O1—C18—H18A	109.5
N39—Ru1—Cl1	84.28 (6)	O1—C18—H18B	109.5
N39—Ru1—Cl2	90.78 (7)	O1—C18—H18C	109.5
N39—Ru1—P1	177.03 (6)	H18A—C18—H18B	109.5
N39—Ru1—N40	79.10 (9)	H18A—C18—H18C	109.5
N39—Ru1—N41	78.59 (9)	H18B—C18—H18C	109.5
N40—Ru1—Cl1	88.54 (7)	C10—C19—H19	119.7
N40—Ru1—Cl2	89.49 (7)	C30—C19—C10	120.6 (3)
N40—Ru1—P1	102.43 (7)	C30—C19—H19	119.7
N41—Ru1—Cl1	93.37 (7)	C11—C20—C15	119.8 (2)
N41—Ru1—Cl2	86.67 (7)	C11—C20—C22	118.6 (2)
N41—Ru1—P1	100.04 (7)	C22—C20—C15	121.6 (2)
N41—Ru1—N40	157.30 (9)	C9—C21—C13	120.6 (3)
O2—P1—Ru1	118.31 (8)	C9—C21—H21	119.7
O2—P1—C10	96.43 (11)	C13—C21—H21	119.7
O3—P1—Ru1	113.14 (8)	C20—C22—H22	120.4
O3—P1—O2	104.16 (10)	C27—C22—C20	119.1 (2)
O3—P1—C10	102.62 (11)	C27—C22—H22	120.4
C10—P1—Ru1	119.52 (9)	N40—C23—C7	122.4 (2)
C16—O1—C18	116.4 (2)	N40—C23—C8	115.4 (2)
C33—O2—P1	120.47 (17)	C7—C23—C8	122.1 (2)
C28—O3—P1	120.35 (17)	O35—C24—C17	111.3 (2)
C24—O35—C32	116.4 (2)	O36—C24—O35	124.3 (3)
C15—O38—C34	116.3 (2)	O36—C24—C17	124.4 (3)
C5—N39—Ru1	118.95 (17)	C8—C25—C17	118.4 (2)
C5—N39—C8	122.3 (2)	C8—C25—H25	120.8
C8—N39—Ru1	118.12 (18)	C17—C25—H25	120.8
C23—N40—Ru1	113.65 (16)	C7—C26—C16	122.2 (2)
C31—N40—Ru1	128.71 (18)	C7—C26—C29	118.7 (2)
C31—N40—C23	117.6 (2)	C29—C26—C16	119.1 (2)
C14—N41—Ru1	115.02 (17)	N41—C27—C22	123.7 (3)
C27—N41—Ru1	127.63 (18)	N41—C27—H27	118.2
C27—N41—C14	117.1 (2)	C22—C27—H27	118.2
N39—C5—C12	120.1 (2)	O3—C28—H28A	109.5
N39—C5—C14	112.8 (2)	O3—C28—H28B	109.5
C12—C5—C14	127.1 (2)	O3—C28—H28C	109.5
C23—C7—H7	120.4	H28A—C28—H28B	109.5
C26—C7—H7	120.4	H28A—C28—H28C	109.5
C26—C7—C23	119.3 (2)	H28B—C28—H28C	109.5
N39—C8—C23	113.1 (2)	C26—C29—H29	120.2
N39—C8—C25	120.3 (2)	C31—C29—C26	119.5 (2)
C25—C8—C23	126.7 (2)	C31—C29—H29	120.2
C21—C9—H9	120.3	C9—C30—H30	119.9
C21—C9—C30	119.3 (3)	C19—C30—C9	120.2 (3)
C30—C9—H9	120.3	C19—C30—H30	119.9
C13—C10—P1	123.1 (2)	N40—C31—C29	122.5 (2)
C13—C10—C19	118.7 (2)	N40—C31—H31	118.7
C19—C10—P1	118.2 (2)	C29—C31—H31	118.7
C14—C11—H11	120.3	O35—C32—H32A	109.5
C14—C11—C20	119.5 (2)	O35—C32—H32B	109.5
C20—C11—H11	120.3	O35—C32—H32C	109.5
C5—C12—H12	120.7	H32A—C32—H32B	109.5
C5—C12—C17	118.6 (2)	H32A—C32—H32C	109.5
C17—C12—H12	120.7	H32B—C32—H32C	109.5
C10—C13—H13	119.7	O2—C33—H33A	109.5
C21—C13—C10	120.6 (3)	O2—C33—H33B	109.5
C21—C13—H13	119.7	O2—C33—H33C	109.5
N41—C14—C5	114.1 (2)	H33A—C33—H33B	109.5
N41—C14—C11	121.9 (2)	H33A—C33—H33C	109.5
C11—C14—C5	123.9 (2)	H33B—C33—H33C	109.5
O4—C15—O38	125.2 (3)	O38—C34—H34A	109.5
O4—C15—C20	124.5 (3)	O38—C34—H34B	109.5
O38—C15—C20	110.3 (2)	O38—C34—H34C	109.5
O1—C16—C26	111.5 (2)	H34A—C34—H34B	109.5
O37—C16—O1	125.0 (2)	H34A—C34—H34C	109.5
O37—C16—C26	123.5 (3)	H34B—C34—H34C	109.5
C12—C17—C24	121.8 (2)	H39A—O39—H39B	105 (4)
			
Ru1—P1—O2—C33	59.1 (2)	C10—P1—O3—C28	46.9 (2)
Ru1—P1—O3—C28	177.04 (17)	C10—C13—C21—C9	−0.6 (4)
Ru1—P1—C10—C13	117.0 (2)	C10—C19—C30—C9	−0.7 (5)
Ru1—P1—C10—C19	−63.9 (2)	C11—C20—C22—C27	−2.4 (4)
Ru1—N39—C5—C12	−171.34 (19)	C12—C5—C14—N41	174.2 (2)
Ru1—N39—C5—C14	7.9 (3)	C12—C5—C14—C11	−7.4 (4)
Ru1—N39—C8—C23	−9.0 (3)	C12—C17—C24—O35	−12.0 (4)
Ru1—N39—C8—C25	170.95 (19)	C12—C17—C24—O36	169.7 (3)
Ru1—N40—C23—C7	177.8 (2)	C12—C17—C25—C8	0.9 (4)
Ru1—N40—C23—C8	−1.0 (3)	C13—C10—C19—C30	0.4 (4)
Ru1—N40—C31—C29	−175.4 (2)	C14—N41—C27—C22	3.2 (4)
Ru1—N41—C14—C5	0.2 (3)	C14—C5—C12—C17	−178.0 (2)
Ru1—N41—C14—C11	−178.35 (19)	C14—C11—C20—C15	−177.4 (2)
Ru1—N41—C27—C22	176.9 (2)	C14—C11—C20—C22	1.7 (4)
P1—C10—C13—C21	179.5 (2)	C15—C20—C22—C27	176.7 (3)
P1—C10—C19—C30	−178.9 (2)	C16—C26—C29—C31	179.0 (2)
O1—C16—C26—C7	−11.2 (4)	C18—O1—C16—O37	−2.6 (4)
O1—C16—C26—C29	169.9 (2)	C18—O1—C16—C26	177.8 (2)
O2—P1—O3—C28	−53.2 (2)	C19—C10—C13—C21	0.3 (4)
O2—P1—C10—C13	−10.8 (2)	C20—C11—C14—N41	1.4 (4)
O2—P1—C10—C19	168.4 (2)	C20—C11—C14—C5	−176.9 (2)
O3—P1—O2—C33	−67.5 (2)	C20—C22—C27—N41	−0.1 (4)
O3—P1—C10—C13	−116.9 (2)	C21—C9—C30—C19	0.4 (4)
O3—P1—C10—C19	62.3 (2)	C23—N40—C31—C29	1.0 (4)
O4—C15—C20—C11	6.8 (4)	C23—C7—C26—C16	−177.2 (2)
O4—C15—C20—C22	−172.3 (3)	C23—C7—C26—C29	1.7 (4)
O37—C16—C26—C7	169.2 (3)	C23—C8—C25—C17	179.7 (2)
O37—C16—C26—C29	−9.8 (4)	C24—C17—C25—C8	−178.0 (2)
O38—C15—C20—C11	−173.4 (2)	C25—C8—C23—N40	−173.6 (2)
O38—C15—C20—C22	7.6 (4)	C25—C8—C23—C7	7.6 (4)
N39—C5—C12—C17	1.1 (4)	C25—C17—C24—O35	166.9 (2)
N39—C5—C14—N41	−5.0 (3)	C25—C17—C24—O36	−11.4 (4)
N39—C5—C14—C11	173.5 (2)	C26—C7—C23—N40	−2.2 (4)
N39—C8—C23—N40	6.3 (3)	C26—C7—C23—C8	176.5 (2)
N39—C8—C23—C7	−172.5 (2)	C26—C29—C31—N40	−1.5 (4)
N39—C8—C25—C17	−0.2 (4)	C27—N41—C14—C5	174.7 (2)
C5—N39—C8—C23	179.9 (2)	C27—N41—C14—C11	−3.8 (4)
C5—N39—C8—C25	−0.2 (4)	C30—C9—C21—C13	0.2 (4)
C5—C12—C17—C24	177.5 (2)	C31—N40—C23—C7	0.9 (4)
C5—C12—C17—C25	−1.4 (4)	C31—N40—C23—C8	−178.0 (2)
C7—C26—C29—C31	0.1 (4)	C32—O35—C24—O36	−1.7 (4)
C8—N39—C5—C12	−0.3 (4)	C32—O35—C24—C17	−179.9 (2)
C8—N39—C5—C14	178.9 (2)	C34—O38—C15—O4	3.9 (4)
C10—P1—O2—C33	−172.3 (2)	C34—O38—C15—C20	−176.0 (2)

### trans-Dichlorido(dimethoxyphenylphosphine)[4,4',4''-tris(methoxycarbonyl)-2,2':6',2''-terpyridine]ruthenium(II) monohydrate Hydrogen-bond geometry (Å, º)

**Table:**

*D*—H···*A*	*D*—H	H···*A*	*D*···*A*	*D*—H···*A*
O39—H39*B*···O36	0.90	2.17	3.00	154.2
O39—H39*A*···Cl1^i^	0.85	2.40	3.24	176.3

Symmetry code: (i) −*x*+1, −*y*+2, −*z*+1.
